# Evolution of swine influenza surveillance and one health governance in Taiwan: from zoonotic risk management to integrated preparedness

**DOI:** 10.1007/s11259-026-11411-0

**Published:** 2026-07-22

**Authors:** Cheng-Han Lin, Tzu-Min Lin, Meng-Wei Lin, Chih-Sheng Lin

**Affiliations:** 1 Department of Biological Science and Technology, National Yang Ming Chiao Tung University, No.75 Bo-Ai Street, Hsinchu, 300 Taiwan; 2 Institute for Information Industry, Digital Transformation Research Institute, Taipei, 100 Taiwan; 3 Center for Intelligent Drug Systems and Smart Bio-devices (IDS2B), National Yang Ming Chiao Tung University, Hsinchu, 300 Taiwan

**Keywords:** Swine influenza surveillance, Swine influenza A (H1N1), One health, Taiwan, Pandemic preparedness, Genomic surveillance

## Abstract

Influenza A viruses (IAVs) remain a persistent global threat because of rapid antigenic change, frequent genetic reassortment, and cross-species transmission. Swine act as key “mixing vessels,” permitting co-infection with avian and human strains and accelerating viral diversification and zoonotic risk. Effective veterinary surveillance is therefore critical for detecting viral evolution within swine populations and for providing early warning at the animal–human interface. Taiwan, home to approximately 5.5 million pigs and 23.4 million people, offers a representative case for examining how swine influenza surveillance has evolved in response to these challenges. This review describes the development of veterinary surveillance strategies from fragmented, event-driven monitoring to a coordinated One Health governance framework incorporating routine whole-genome sequencing, targeted serological surveillance, and coordinated data exchange across sectors. Key historical milestones include mass vaccination and culling programs, the 2009 pandemic response and recent zoonotic detections of H1N2v in humans (2021–2023), collectively evidencing ongoing bidirectional viral exchange. Episodes of severe human seasonal influenza activity, including H3N2-associated outbreaks, further highlighted the importance of coordinated surveillance across human and animal health sectors. By focusing on surveillance practices applied to animals, this review highlights the practical value of integrating molecular epidemiology, veterinary field monitoring, artificial intelligence (AI)-enabled technologies and cross-sector communication under a One Health framework. The Taiwanese experience demonstrates how strengthened veterinary surveillance can improve the sensitivity for detecting reassortment events, enhance traceability of viral spread, and support timely risk assessment in intensive livestock systems. These lessons offer a practical, replicable pathway for other countries aiming to overcome swine influenza surveillance fragmentation and strengthen pandemic preparedness under a One Health framework.

**Graphical Abstract:**

**Figure:**
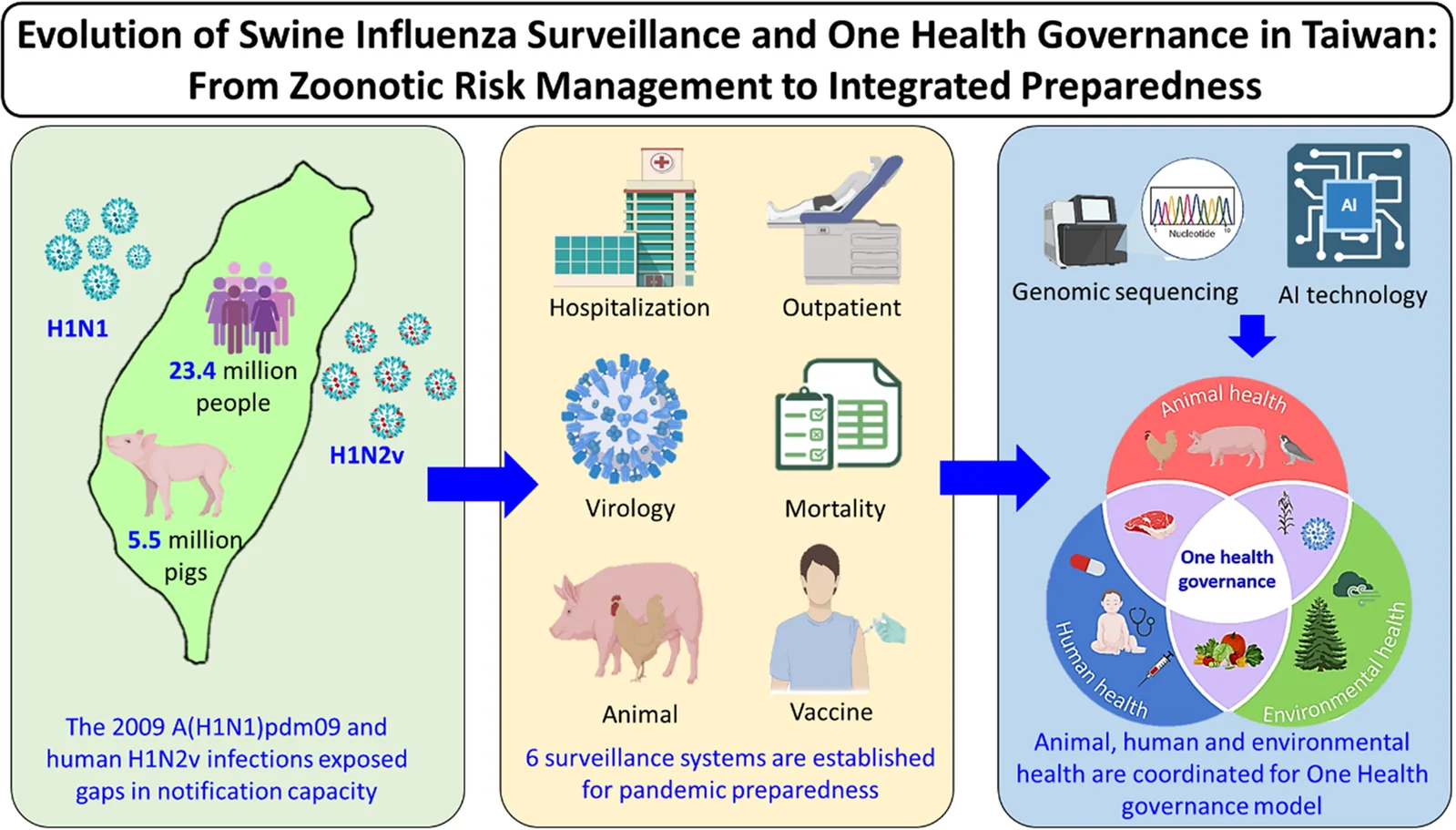
The review traces Taiwan’s surveillance evolution from fragmented monitoring of swine influenza A viruses to a coordinated One Health governance model. A(H1N1)pdm09 and human H1N2v infections exposed gaps in notification capacity. The surveillance systems, genome sequencing and AI-enabled analytics are established to link animal, human, and environmental health for pandemic preparedness.

## Introduction

Emerging influenza A viruses represent a persistent challenge for veterinary health because of their extensive genetic diversity, frequent reassortment, and continuous circulation (Korsun et al. 2025; Liang et al. 2012). Swine are pivotal “mixing vessels” because their respiratory tracts co-express α2,3- and α2,6-linked sialic acid receptors, permitting simultaneous infection by avian and human influenza strains and enabling genetic reassortment (Mancera Gracia et al. 2020; Kristensen et al. 2024). Given cross-species transmissibility, swine have emerged as critical intermediate hosts in the ecology and evolution of influenza A viruses (Er 2025). Pork is the world’s most consumed meat, representing 34% of global meat intake; annual consumption is approximately 124 million tons and the sector’s value exceeds USD 350 billion (FAO 2024a). More than 700 million swine create dense interfaces between humans and livestock, increasing opportunities for zoonotic spillover (Gong et al. 2018a). The 2009 H1N1 pandemic, caused by a swine-origin virus, exemplified the pandemic potential of these interfaces (Assavacheep and Thanawongnuwech 2022). The pandemic led to economic losses from production setbacks, movement restrictions, and trade suspensions that reached an estimated USD 3 billion (de Courville et al. 2022). Influenza circulation has reduced pork-sector profitability in global swine-production settings by roughly 10% (Moraes et al. 2023). Production contractions in parts of Asia have left surveillance systems fragmented and sector-specific, with poor integration across veterinary, human, and environmental domains. This fragmentation delays detection and imposes measurable economic costs estimated at USD 1–5 per pig annually across a > 700-million-head industry (Moraes et al. 2023). Therefore, swine influenza represents both a zoonotic threat and an economic destabilizer of the global pork supply chain, underscoring the need for integrated One Health surveillance to protect public health and agricultural economies.

Taiwan exemplifies the intersection of intensive pig production and human influenza dynamics in East Asia. With approximately 5.5 million pigs and 23.4 million people, dense human–swine interfaces increase opportunities for viral reassortment and zoonotic spillover (Hui et al. 2025). Since 2009, H1N1 and H3N2 influenza strains have remained recurrent public-health burdens in Taiwan; the Taiwan Centers for Disease Control (TCDC) reported 1,663 laboratories-confirmed severe human infections and 163 deaths, underscoring the health burden of seasonal and pandemic influenza activity (TCDC 2016). Although annual influenza vaccination campaigns in the human population reach approximately 40% coverage, antigenic drift, particularly in H3N2, continues to limit vaccine effectiveness and complicate control efforts (Trevisan et al. 2021). Beyond human health, influenza and other infectious diseases impose substantial economic losses on the livestock sector. Taiwan produces an estimated 8–9 million pigs annually, contributing about USD 2.5 billion to the agricultural economy (Republic of China Swine Association 2025). Herd-level outbreaks reduce growth rates and feed conversion efficiency and increase mortality, with typical production losses of about 5% per outbreak cycle; routine vaccination and basic biosecurity measures cost roughly USD 4 per pig per year (Moraes et al. 2023). Previous major livestock-disease events in Taiwan involved the culling of approximately 3.85 million pigs and suspension of pork exports, demonstrating the economic vulnerability of the swine sector and the importance of sustained animal-health surveillance. Taiwan faced embargoes and emergency slaughtering that produced short-term shocks to supply chains and farm incomes. These experiences demonstrate that effective influenza control in Taiwan requires more than episodic responses. Continuous vaccination and clinical surveillance must be complemented by integrated One Health monitoring that links veterinary, human-health, and environmental surveillance, thereby reducing the risk of cross-species transmission and protecting both public health and agricultural stability.

This review traces the evolution of Taiwan’s swine influenza surveillance from a fragmented set of sectoral programs to a coordinated One Health governance model. Taiwan consolidated a cross-sector surveillance framework that links the Bureau of Animal and Plant Health Inspection and Quarantine (BAPHIQ), the Animal Health Research Institute (AHRI), and the TCDC to strengthen early warning and pandemic preparedness (Gong et al. 2018b). Over the past two decades, the country has shifted from predominantly reactive responses toward an integrated surveillance ecosystem that synthesizes genomic, clinical, veterinary, and environmental data streams (Yang et al. 2018). This article situates Taiwan’s experience within global influenza prevention and control efforts, illustrating how technological innovation, cross-departmental coordination, and adaptive policy reforms have improved outbreak detection, response speed, and situational awareness. By examining the operational interfaces among veterinary epidemiology, human-health surveillance, and environmental monitoring, the review highlights the strategic value of Taiwan’s model and argues that its governance architecture and data-integration practices offer a replicable pathway for other countries seeking to overcome surveillance fragmentation and strengthen pandemic readiness under a One Health framework.

## Global swine influenza surveillance systems and governance context

Swine influenza A poses a persistent global challenge because genetically diverse lineages can persist in pig populations and undergo episodic reassortment, occasionally generating viruses with altered antigenic properties and zoonotic potential. The virus’s origins in North America and European Union highlighted the need for sustained internationally coordinated surveillance. Multiple variant influenza viruses have since been detected in humans, prompting integration of animal and public-health surveillance systems. Global collaboration is facilitated by the World Organization for Animal Health (WOAH), Food and Agriculture Organization (FAO), and World Health Organization (WHO) through the OIE/FAO Network of expertise on animal influenza (OFFLU) network, which enables the exchange of genomic, epidemiological, and risk-assessment data.

### European Union (EU)

Swine influenza has circulated enzootically in Europe since the late 1970 s, originating from avian-derived introductions and diversifying within swine populations. Subsequent reassortment in the 1990 s generated H3N2 and H1N2 variants, including human-seasonal HA introductions on swine internal gene backgrounds (clade 1B) (Trinh et al. 2020). During the 2009 A(H1N1)pdm09 pandemic, more than 500,000 laboratory-confirmed human cases and approximately 7,000 human deaths were reported, amplified reassortment dynamics and established the H1N1pdm09 internal gene cassette as a dominant backbone in many European swine viruses (Amato Gauci et al. 2010). Following 2009, H1N1 triggered extensive secondary reassortment, generating multiple reassortant H1N1, H1N2, and H3N2 swine influenza virus genotypes in European pig populations and establishing the H1N1pdm09 internal gene cassette as a dominant backbone, particularly in the United Kingdom (Moraes et al. 2022). In the European Union, swine influenza surveillance is supported by member-state activities, research networks, and collaborative molecular epidemiology initiatives. However, routine swine influenza surveillance is not uniformly harmonized across all member states, and surveillance intensity varies among countries. European collaborative networks and risk-assessment bodies provide useful frameworks for data interpretation and communication, but they should not be described as a fully harmonized EU-wide routine surveillance system for swine influenza viruses (Bronzwaer et al. 2022). Continuous sequencing efforts in Germany, France, and the United Kingdom produce high-resolution phylogenetic maps that inform OFFLU reporting and contribute to vaccine-strain selection, making EU a model for cross-border surveillance.

### North America

Classical swine H1N1 (clade 1 A), historically derived from the 1918 lineage, remained predominant in North America swine populations until the late 1990 s (Stern et al. 2010), when triple-reassortant H3N2 viruses emerged and accelerated viral diversification (Chepkwony et al. 2021). The 2009 H1N1pdm09 episode further reshaped viral ecology through extensive secondary reassortment, producing multiple H1N1 and H1N2 lineages (α, β, γ, and δ clades). From April 12, 2009 to April 10, 2010, the Centers for Disease Control and Prevention of USA (CDC) estimated there were 60.8 million cases and 12,469 deaths in the USA due to the (H1N1)pdm09 virus (CDC 2010). In North America, structured swine influenza surveillance is most extensively documented in the United States, where United States Department of Agriculture (USDA), CDC collaboration, and the National Animal Health Laboratory Network (NAHLN) combine active and passive sampling across farms, slaughterhouses, and diagnostic submissions (Thomas et al. 2025). Broad uptake of genomic sequencing, supported by public databases, enables fine-scale molecular epidemiology and informs vaccine development, positioning North America as a benchmark for preparedness and interagency coordination.

### Asia

Asia, which contains more than half of global swine biomass, exhibits exceptional influenza A virus diversity in swine. Classical swine H1N1 persisted into the late twentieth century before admixture with European and American lineages through trade and animal movement. Post-2010 introductions of A(H1N1)pdm09 generated a heterogeneous viral ecosystem in which multiple H1 (clades 1A–1C) and H3 clades co-circulate alongside episodic avian-origin reassortants, e.g., H5N1 and H9N2 (Roy et al. 2025; Yu et al. 2025). Surveillance intensity, sequencing capacity, and public accessibility of sequence data vary markedly across countries, impeding comprehensive regional situational awareness. The Asia–Pacific region includes distinct epidemiological settings; for example, Australia’s geographic isolation produced locally adapted swine lineages that now co-circulate with A(H1N1)pdm09 and form antigenically distinct populations.

### Comparative summary

Regional differences in lead agencies, primary data platforms, and system strengths shape surveillance performance. These international contexts illustrate that surveillance systems differ in institutional design, sampling intensity, genomic capacity, and data-sharing practices. The purpose of this comparison is not to rank systems, but to clarify how surveillance model has evolved in relation to selected global surveillance approaches (Table 1).

**Table 1:** Comparative overview of national and regional swine influenza surveillance systems

Attribute	Asia	North America	European Union (EU)
Index year (first documented swine-origin case or formal surveillance start)	1998	1918 Modern organized surveillance from late twentieth century	1979 Regional coordination initiatives began
Lead agencies and partners	BAPHIQ, AHRI, TCDC	USDA, CDC, NAHLN	EFSA, ECDC
Primary data platforms and sharing	Whole-genome sequences and National surveillance databases	Influenza Research Database and GenBank	High transparency and cross-border data sharing
Zoonotic-event detection and reporting practice	Documented human H1N2v cases in 2021–2023 with WHO/IHR notifications	Multiple confirmed variant cases (H1N1v, H3N2v) annually	Rare zoonotic detections documented in annual EFSA–ECDC reports
System strengths	High-resolution genomic surveillance; rapid WHO/IHR notification pathways	Large-scale, standardized, and data-rich model	Harmonized protocols and continent-wide data comparability
Limitations	Constrained staffing and operational resources relative to workload	High operational cost and uneven private-sector participation	Dependence on member-state reporting and occasional data lag
References	Gong et al. 2018b Yang et al. 2018 Roy et al. 2025 Yu et al. 2025	CDC 2010 Chepkwony et al. 2021 Stern et al. 2010	Bronzwaer et al. 2022 Trinh et al. 2020

## Historical surveillance and major swine influenza events in Taiwan

**Fig. 1:**
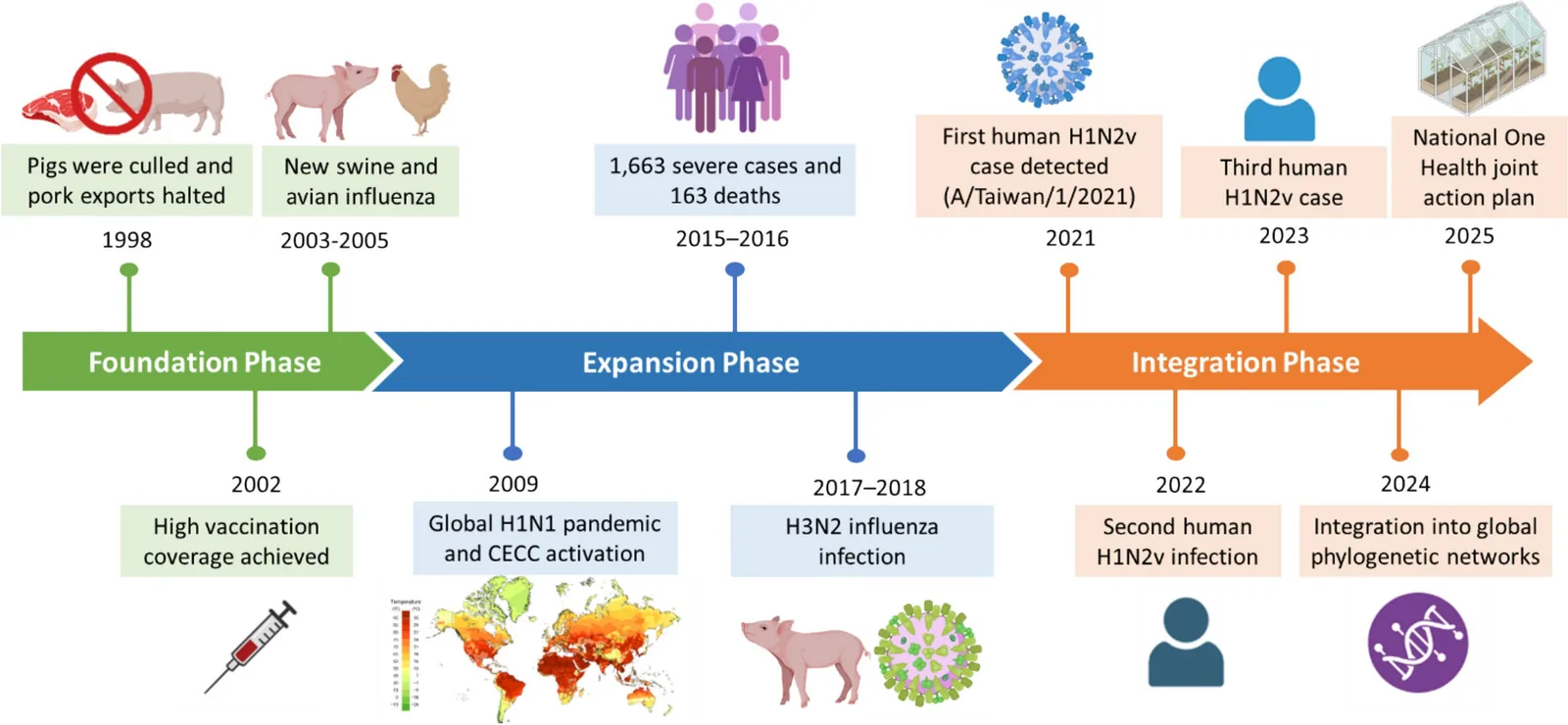
Timeline of influenza surveillance, major events, and integration actions in Taiwan. The diagram illustrates the key phases and milestone events from the Foundation Phase, through the Expansion Phase to the Integration Phase, illustrating the evolution of influenza surveillance, outbreak response, and One Health integration in Taiwan currently. The schematic diagram was created by the authors using BioRender.com (BioRender, Toronto, ON, Canada). Created in BioRender by Lin (2026). https://BioRender.com/n8c6tsl

Swine influenza A virus has been under active surveillance in Taiwan for more than two decades. Given the country’s dense pig population and its integration into regional livestock trade networks, Taiwan occupies a strategic position for monitoring viral evolution at the animal–human interface (Fig. 1). Continuous molecular characterization has documented endemic swine lineages and recurrent reassortment involving human- and avian-origin influenza gene segments. These findings underscore the importance of sustained genomic surveillance.

### Early foundation phase of swine influenza monitoring (1998–2008)

Nationwide surveillance for swine influenza began in the late 1990 s, coordinated by the BAPHIQ and the AHRI under the Ministry of Agriculture (MOA). Taiwan implemented the most comprehensive epizootic-control strategy that included mass vaccination and culling programs. More than 3 million pigs were eliminated within 4 months (MOA 2002). Vaccination coverage reached 87%, and neutralizing antibody titers exceeded protective thresholds in more than half of the tested animals by 2002 (MOA 2022a). Routine nasal swabs, respiratory tissue samples, and serum sampling from sentinel farms and slaughterhouses enabled both virological detection and seroepidemiological mapping across Taiwan. In 2003, researchers isolated and characterized a novel H3N1 swine influenza virus from local pig herds, marking the first recorded emergence of a reassortant subtype in Taiwan (Tsai and Pan 2003). This strain carried an H3 gene of human seasonal origin and an N1 gene derived from the classical swine lineage, suggesting active gene exchange within Taiwanese pig populations. The multiple H1N1 variants with distinct antigenic profiles indicate co-circulation of diverse viral populations within commercial farms.

### The 2009 H1N1 pandemic (2009–2010)

A swine-origin triple reassortant comprising North American and Eurasian gene segments caused the 2009 influenza A(H1N1)pdm09 pandemic. This event marked a pivotal inflection point for Taiwan’s influenza preparedness (Table 2). The TCDC activated the Central Epidemic Command Center (CECC) to coordinate actions across health, agriculture, education, and transportation sectors. The 1 st severe human case and 1 st human death prompted the National Health Insurance Administration (NHIA) to expand reimbursement for laboratory-confirmed influenza A cases (TCDC 2009a). Key interventions included border screening, rapid case reporting, laboratory confirmation, and “3–2–5” school-suspension non-pharmaceutical interventions. The government also launched a national H1N1 vaccination program for healthcare workers, schoolchildren, and high-risk groups (TCDC 2009b).

**Table 2:** Major public-health events and actions during the 2009 H1N1 pandemic in Taiwan

**D**ate (2009)	Event/public-health action
April	• Following the WHO's elevation of the pandemic alert to Phase 4, Taiwan's government activated the CECC to coordinate national response efforts • Taiwan implemented border quarantine with fever screening at airports to delay the viral entry
**May**	• CECC formalized an H1N1 epidemic response tier "H1N1 Influenza Epidemic Level Table" • The first laboratory-confirmed H1N1 case was reported domestically, prompting elevation of the national epidemic level, enhanced surveillance and contact-tracing activities
June–August	• The 1 st severe case 1 st death case of H1N1 influenza were discovered • The 2nd death case of H1N1 influenza was reported • The NHIA expanded reimbursement for neuraminidase inhibitors • The CECC adopted the “3–2–5” school-closure standard (≥ 2 confirmed cases in a class within 3 consecutive days → 5-day suspension)
September	• Virologic surveillance indicated H1N1 predominance: over 90% of community influenza A isolates characterized as H1N1, signaling sustained community transmission and shaping vaccine and clinical management priorities
October–December	• A 7-year-old child died of sepsis after receiving the new influenza vaccine. CECC has released a database of adverse reaction cases • Taiwan launched a mass vaccination campaign, prioritizing healthcare workers, schoolchildren, and high-risk groups. The campaign aimed to immunize millions to curb community spread
2010 and beyond	• Pandemic H1N1 virus was incorporated into routine seasonal influenza surveillance and vaccine strain selection. Post-pandemic evaluations informed revisions to national preparedness plans, surveillance algorithms, and cross-sector coordination mechanisms

However, the campaign faced public scrutiny after a 7-year-old child died from sepsis following vaccination. The campaign generated public concern regarding crisis communication and lack of transparency, alleging administrative errors and protection of corporate interests (TCDC 2009c). Between May 2009 and February 2010, Taiwan reported over 3,000 laboratory-confirmed A(H1N1)pdm09 cases and 46 deaths, corresponding to an approximate mortality rate of 1.8 per million (Hsueh et al. 2010). Serologic data indicated widespread exposure, particularly among school-aged children and young adults. The National Influenza Vaccination Program achieved 25% population coverage by early 2010 (Gong et al. 2018b). Retrospective veterinary surveillance identified H1N1pdm09 internal gene segments in swine isolates collected after the human pandemic peak. These findings provided direct evidence of human-to-swine reverse zoonosis (Yen et al. 2014). This spillover event established the H1N1pdm09 internal gene cassette as a recurrent backbone in Taiwan’s swine influenza A viruses, shaping the genetic architecture of subsequent reassortant strains.

### Evidence of cross-species transmission and viral diversity (2011–2018)

Following the 2009 H1N1 pandemic, Taiwan significantly intensified surveillance to monitor potential human–swine transmission. Retrospective analyses revealed introductions of H1N1pdm09-like internal genes into local swine influenza viruses, consistent with regional patterns observed across East Asia (Gong et al. 2018b). Between 2011 and 2018, Taiwan maintained active monitoring of swine influenza A viruses and several large-scale seasonal influenza outbreaks in humans, underscoring the linked viral evolution across species.

The 2015–2016 season marked the most severe post-pandemic A(H1N1)pdm09 wave in Taiwan. TCDC reported 1,663 laboratory-confirmed severe infections and 163 deaths (TCDC 2016). Clinical presentations included multi-organ complications and increased demand for intensive care unit (ICU) resources. In parallel, MOA-affiliated laboratories continued whole-genome sequencing of swine isolates and detected Eurasian avian-like H1 lineage signatures reassorted with pdm09 internal gene cassettes. These data indicated ongoing reassortment within domestic herds.

Between 2016 and 2018, human seasonal influenza A(H3N2) activity became an important component of Taiwan’s influenza surveillance landscape. This H3N2 activity primarily referred to human clinical surveillance rather than the emergence of a newly documented swine H3N2 lineage in Taiwan. During the 2016–2017 season, Taiwan experienced an unusually prolonged influenza epidemic, and increased viral fitness, reduced population immunity, and climatic factors were suggested to have contributed to this extended activity rather than a single major antigenic shift (Yang et al. 2018). Between October 2017 and March 2018, influenza viruses caused 610 severe human infections and 88 deaths in Taiwan, further demonstrating the public-health burden of seasonal influenza and the need for continuous virological monitoring (TCDC 2018a). Although these H3N2-associated events were documented mainly through human surveillance systems, they also reinforced the importance of maintaining parallel veterinary surveillance because swine populations can sustain influenza A virus reassortment and may serve as an interface for bidirectional transmission. In response to broader concerns regarding influenza virus evolution, zoonotic risk, and animal-health preparedness, Taiwan strengthened slaughter inspection protocols and farm-level prevention measures. All pigs were subject to pre- and post-slaughter inspection by BAPHIQ personnel, and farmers were encouraged to maintain vaccination practices, improve farm biosecurity, and adopt basic hygiene measures to reduce respiratory disease transmission and potential cross-species risk (AHRI 2018). These measures were implemented primarily as preventive and preparedness actions; publicly available reports do not provide a direct quantified estimate of swine influenza cases detected specifically through these strengthened slaughter inspection measures.

### Integration phase of genomic surveillance and human H1N2v cases (2021–2025)

A milestone in Taiwan’s influenza surveillance occurred in 2021, when the first human case of swine-origin influenza A(H1N2)v infection was confirmed in a 5-year-old girl (Yang et al. 2025). The A/Taiwan/1/2021 (H1N2) isolate carried HA and NA genes from endemic Taiwanese swine H1N2 viruses and internal genes from human H1N1pdm09. Genome sequencing indicated bidirectional viral exchange between humans and pigs. Phylogenetic analysis placed the isolate within a distinct Taiwanese clade and supported localized viral evolution (Hui et al. 2025). This event prompted enhancements in national genomic surveillance and reinforced Taiwan’s participation in the Global Initiative on Sharing All Influenza Data (GISAID). In October 2022, TCDC confirmed a 2nd human H1N2v infection in a 7-year-old girl from central Taiwan whose family was engaged in livestock farming (TCDC 2022). The patient presented with fever, myalgia, anorexia, cough, and rhinorrhea. Genetic sequencing revealed an H1N2v strain closely related to local swine isolates, suggesting independent viral evolution (Taipei Times 2022). The event was classified as a zoonotic infection and reported to the WHO under the IHR framework. In March 2023, TCDC reported a 3rd human case of swine-origin influenza A(H1N2v) infection in a teenage girl from central Taiwan with recent pig exposure (TCDC 2023). Genomic sequencing confirmed H1N2v and showed similarity to the 2021 isolate but distinct from prior strains, indicating independent zoonotic spillover. This event further demonstrated Taiwan’s One Health-based surveillance capacity, integrating human, animal, and environmental monitoring to detect emerging influenza variants.

The H1N2v events in 2021–2023 demonstrated the continued circulation of swine-origin H1N2 viruses with zoonotic potential in Taiwan and validated One Health surveillance for detecting rare spillover events. By 2024, Taiwan’s swine influenza isolates were integrated into Microreact and HA clade mapping systems, enabling comparative analysis with regional strains (Li et al. 2025). Antigenic characterization revealed partial mismatches between local H1 lineages and existing candidate vaccine viruses. The findings prompted discussion on region-specific vaccine design. Taiwan’s surveillance data were officially incorporated into the 2024 OFFLU biannual report and reflected continued regional contribution (Hui et al. 2025). Risk reduction would focus on targeted serologic surveillance of high-exposure workers in farms. Next-generation sequencing combined with rapid data sharing is enable real-time phylogenetic tracking. Vaccine antigenic matching and periodic biosecurity audits will mitigate viral adaptation and lower spillover risk.

## National notifiable disease surveillance reform following the pandemic

The 2009 influenza A(H1N1)pdm09 pandemic marked a pivotal transition in Taiwan’s infectious disease surveillance infrastructure. The crisis accelerated the shift from a fragmented, event-driven system to a fully integrated nationwide network. Following the 2003 severe acute respiratory syndrome (SARS) outbreak, Taiwan strengthened its legal notification framework and classified influenza as a Category I notifiable disease for emerging infectious diseases (Wang et al. 2007). During the early pandemic-preparedness period, novel influenza was managed under a high-alert notification model to support rapid detection and emergency response. However, the 2009 A(H1N1)pdm09 pandemic showed that broad notification of influenza-like illness could overload reporting systems and reduce the efficiency of severity-based risk assessment. Taiwan therefore shifted toward a Category IV notification framework for severe complicated influenza, in which reporting emphasized laboratory-confirmed severe human cases, hospitalization, intensive-care use, and mortality rather than all mild influenza-like illness. This reclassification improved prioritization of clinically significant cases and supported more efficient allocation of public-health and medical resources (TCDC 2012). This reform established the foundation for 6 major surveillance architectures: hospitalization, virological, outpatient, mortality, animal and vaccine surveillance.

### Hospitalization surveillance system

The hospitalization surveillance system became the cornerstone for assessing disease severity and managing healthcare capacity. By consolidating multiple reporting channels into a unified electronic portal, hospitals and public health officers could promptly report all laboratory-confirmed severe influenza cases in real time (Yang et al. 2018). Each record includes demographic data, admission dates, clinical manifestations, comorbidities, treatment regimens, and use of intensive-care resources such as extracorporeal membrane oxygenation and intravenous immunoglobulin (Chou et al. 2020). Local health bureaus update records daily and regional TCDC branches oversee quality control. This system enables real-time analysis of hospitalization trends, fatality ratios, and high-risk subgroups to support allocation of ICU beds and antiviral supplies during epidemic peaks (Kao et al. 2009).

### Virological surveillance system

Taiwan’s virological surveillance system expanded significantly after the H1N1 pandemic. Before 1999, only a few laboratories were capable of viral diagnostics. In response, TCDC established the Contract Viral Infection Laboratory Network, which grew to 10 regional laboratories by 2009. During the pandemic, these laboratories collected specimens from influenza-like illness (ILI) cases within 3 days of symptom onset and reported daily positivity rates (Yang et al. 2025). These data enabled continuous assessment of community-level influenza activity and the prevalence of novel H1N1 strains (Chuang et al. 2012). Concurrently, the National Influenza Center launched an antiviral-resistance monitoring program. Integration of subtyping data, resistance profiles, and NHIA patient-visit records improved estimation of community infection levels and informed vaccine strain selection and antiviral policy. These developments formed the basis of Taiwan’s genomic surveillance infrastructure.

### Outpatient surveillance system

The outpatient surveillance system integrates the Real-time Outbreak and Disease Surveillance (RODS) platform with the NHI smart-card database to monitor community-level trends (Lin et al. 2018). Originally developed at the University of Pittsburgh and introduced to Taiwan in 1999, RODS collect emergency department data from participating hospitals through automated uploads of ICD diagnostic codes. By 2009, approximately 160 emergency departments (covering 80% of hospitals) were transmitting 90,000 records weekly. These data generate real-time visualizations of respiratory syndrome trends stratified by hospital level. The addition of NHI outpatient visit data captures mild ILI consultations. Cross-validation with the sentinel-physician surveillance program demonstrated a high correlation with weekly trends and confirmed the system's sensitivity and stability (Lai et al. 2010). This integration enables forecasting of epidemic activity approximately 1 week in advance and supports non-pharmaceutical interventions such as Taiwan’s “3–2–5” school-closure standard.

### Mortality surveillance system

To enhance fatality assessment and improve detection of excess deaths, TCDC established the Surveillance Pneumonia and Influenza system through the National Death Certificate System in 2009 (TCDC 2009d). Unlike systems in many countries that rely solely on finalized cause-of-death statistics, Taiwan’s approach links mortality monitoring directly with the notifiable disease database. When a severe influenza case leads to death, the system sends an automatic alert to public health officers for rapid verification. Simultaneously, the online death-reporting system of TCDC extracts records containing keywords such as “pneumonia,” “cold,” or “influenza,” calculates weekly death counts, and applies a 4-week moving average to smooth short-term fluctuations. Statistical analysis shows a high correlation between this real-time system and finalized annual records for an early indicator of epidemic severity (Lai et al. 2010).

### Animal surveillance system

Taiwan’s animal surveillance system, led by BAPHIQ and AHRI, has adopted advanced Internet-of-Things (IoT) technologies to improve monitoring capacity. In poultry, comb-color recognition algorithms, high-resolution imaging, and virtual-reality coops serve as supplementary health indicators. In swine, Radio Frequency Identification (RFID) system was linked to digital animal-management platforms that track productivity metrics and movement histories. AI-based image recognition of lesions and pneumonia is being evaluated as a point-of-care triage tool for respiratory disease (MOA 2022b). Slaughterhouses have deployed digital hygiene inspection and remote video oversight in 16 HACCP-certified abattoirs and 9 major pork markets, covering 50% of national pig slaughter (4 million head) and 45% of poultry (150 million birds) (MOA 2025). These systems are connected to cloud-based dashboards with micro-weather and environmental sensors for on-site anomaly detection and workload optimization.

### Vaccine surveillance system (2010–2025)

Taiwan’s vaccine surveillance system monitors coverage, safety, and effectiveness of seasonal influenza vaccines in humans and preventive immunization programs in livestock. For human influenza, TCDC integrates data from the NHI smart-card system, the National Immunization Information System (NIIS), and sentinel hospitals to track uptake and lot-specific adverse events (TCDC 2014). Each vaccination record is linked to clinical data and contributes to weekly dashboards by age, county, and priority group. The vaccine program has achieved 40–45% population coverage and vaccine effectiveness has been evaluated using hospitalization data (TCDC 2025a). Post-marketing surveillance uses automated barcode scanning for cold-chain management and lot tracking at healthcare facilities. For livestock, BAPHIQ and AHRI oversee mandatory vaccination campaigns for swine and poultry. Coverage and batch data are reported by local animal disease control centers and uploaded to BAPHIQ’s disease prevention network. Import and export quarantine declarations are processed through a cloud-based single-window platform (ARHI 2021). Surveillance design follows WOAH standards that define target populations, sampling frames, and laboratory workflows. Statistical analysis of serologic and virologic data supports population-level infection trend estimation and early intervention (BAPHIQ 2025).

Together, these 6 surveillance systems form a multilayered and complementary network that integrates hospitalization, virological, outpatient, mortality, animal, and vaccine surveillance into a unified framework (Table 3). This multi-source integration enables cross-verification of signals, real-time adjustment of risk assessments, and rapid policy response. The H1N1 pandemic served not only as a public health challenge but also as a catalyst for the modernization of Taiwan’s influenza surveillance. The development transformed short-term crisis management into a sustained, data-driven mechanism for early warning, preparedness, and decision-making. These reforms also facilitated integration of human and animal influenza data, which is central to Taiwan’s One Health approach to swine influenza surveillance.

**Table 3:** Structured presentation of the National One Health joint action plan strategic objectives in Taiwan

Influenza A surveillance	System	Core data & indicators	Operational use	References
Hospitalization	TCDC notifiable disease platform	**Core data:** Demographics, onset, admission, discharge, comorbidities, treatment **Indicators**: hospitalizations, ICU occupancy, age-risk profile	Severity assessment, ICU surge planning, antiviral allocation	Chou et al. 2020 Yang et al. 2018
Virological	Lab network + National Influenza Center	**Core data:** antigenic analysis and ILI cases **Indicators**: positivity, subtype, resistance frequency	Track community viral activity and detect emergence/reassortment	Chuang et al. 2012 Yang et al. 2025
Outpatient	RODS + NHI smart-card	**Core data:** International Classification of Diseases **Indicators:** ILI rate, 160 hospital emergency departments	Earliest community signal; about 1-week forecasting (3–2–5 school closure)	Lai et al. 2010 Lin et al. 2018
Mortality	Real-time surveillance of pneumonia and influenza in the National Death Certificate System	**Core data:** Cause-of-death text (pneumonia/influenza) **Indicators:** Pneumonia and Influenza deaths, excess-mortality signal	Early detection of excess deaths, severity estimates and risk communication	TCDC 2009d Lai et al. 2010
Animal health	Agricultural internet of things development plan	**Core data:** Farm reports, lab confirmations, RFID **Indicators:** Species clusters, abnormal mortality, genomic links to human strains	Cross-verification; movement control, targeted sampling	MOA 2022b MOA 2025
Vaccine	Human: NIIS and NHI; Livestock: BAPHIQ and the AHRI	**Core data:** Weekly dashboards by age, county, and priority groups; **Indicators:** Vaccine percentage, breakthrough rates and farm-level immunity profiles	Targeted campaigns, detect waning or antigenic mismatch; verify livestock program performance	ARHI 2021 BAPHIQ 2025 TCDC 2014 TCDC 2025a

## One health governance against influenza pandemics in Taiwan

### Cross-sectoral collaboration following influenza pandemics

The influenza pandemics exposed critical gaps in surveillance across human, animal, and environmental health systems to improve cross-sectoral coordination in Taiwan. Building on these lessons, Taiwan aligns its policies with the One Health High-Level Expert Panel (OHHLEP). The framework was endorsed by the WHO, WOAH, and the FAO (WHO 2022). Current priorities include policy coherence, shared indicators, and joint prevention strategies across sectors. This initiative seeks to translate global guidance into national operations, with a focus on early warning and rapid response to high-impact zoonoses, including highly pathogenic avian influenza and swine influenza A viruses (OHHLEP et al. 2022).

### A unified approach to optimizing the health of people, animals, and ecosystems

**Fig. 2:**
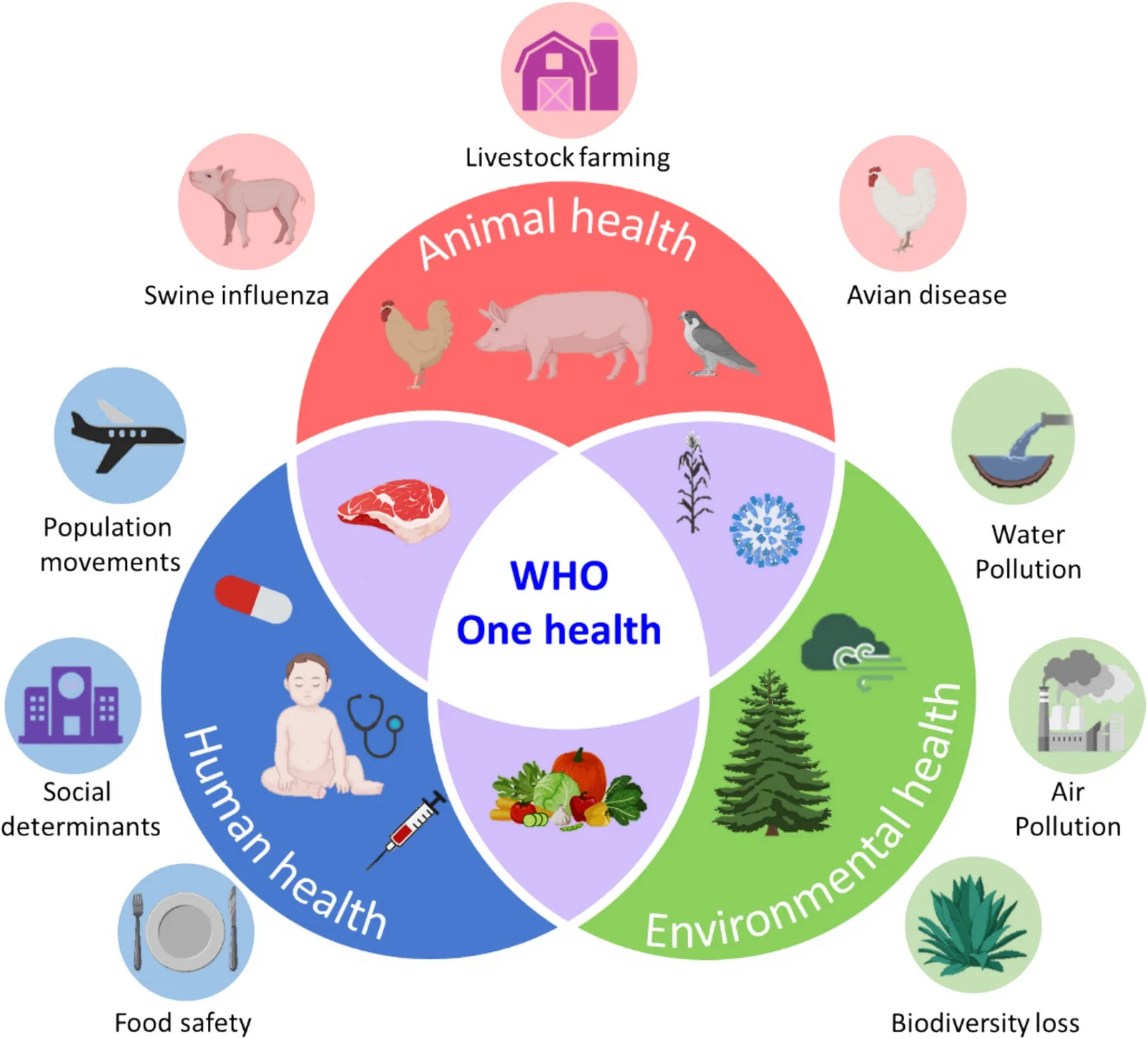
One Health framework linking environmental, animal, and human drivers of swine influenza. Schematic diagram showing how the One Health approach connects Animal health, Human health, and Environmental health to the emergence and spread of swine influenza. The central concept One Health (often represented at the diagram’s core) integrates actions and information across sectors and is supported by global public health leadership (WHO). The schematic diagram was created by the authors using BioRender.com (BioRender, Toronto, ON, Canada). Created in BioRender by Lin (2026). https://BioRender.com/n8c6tsl

One Health is a collaborative, multisectoral, and transdisciplinary approach that recognizes the interdependence of human, animal, and environmental health in achieving optimal health outcomes (Fig. 2). It mobilizes stakeholders across disciplines, sectors, and communities to promote well-being and address complex health threats such as zoonotic diseases, food insecurity, and the impacts of climate change (FAO 2024b). The One Health framework extends beyond zoonoses and antimicrobial resistance (AMR) to encompass prevention, health promotion, crisis preparedness, response, and recovery (CDC 2025).

The One Health approach is guided by five core principles:

- **Multidisciplinary collaboration:** This principle emphasizes the integration of expertise across human medicine, veterinary science, environmental health, and social sciences. This principle requires equitable participation of different professions in health governance.
- **Socio-political and multicultural parity:** One Health upholds the principle that all individuals and communities should have equal rights, opportunities, and representation. It promotes meaningful engagement of affected populations in health decision-making, particularly marginalized and underrepresented groups.
- **Socioecological equilibrium:** This principle seeks a harmonious balance among human, animal, and environmental systems. It recognizes the intrinsic value of biodiversity, equitable access to natural resources, and the importance of preserving ecological integrity.
- **Stewardship and human responsibility:** One Health calls for sustainable behaviors and policies that safeguard animal welfare and ecosystem health. It highlights intergenerational responsibility and the need to protect the well-being of current and future generations.
- **Transdisciplinary and multisectoral integration:** This principal advocates for the inclusion of diverse knowledge systems, including scientific, indigenous, and local perspectives. It encourages coordinated action across public health, agriculture, environment, and other sectors to design culturally sensitive and effective solutions.

Together, these principles form the foundation of Taiwan’s evolving One Health governance model, which aims to strengthen pandemic preparedness, enhance interagency coordination, and promote resilience across human and animal health systems.

### National one health joint plan of action (2026–2030)

The TCDC concluded that recent pandemics exposed the systemic and interdependent challenges linking human health, animal health, and ecosystem integrity. Addressing these challenges requires a nationally led, integrated action plan that operationalizes the One Health approach. In March 2025, the Executive Yuan of Taiwan mandated acceleration of the National One Health Joint Plan of Action (TCDC 2025b). Taiwan formally adopted a One Health framework and established a cabinet-level interagency mechanism that includes the Ministry of Health and Welfare, the Ministry of Agriculture, the Ministry of Environment, and the Ministry of the Interior (Ministry of Health and Welfare 2025).

**Fig. 3:**
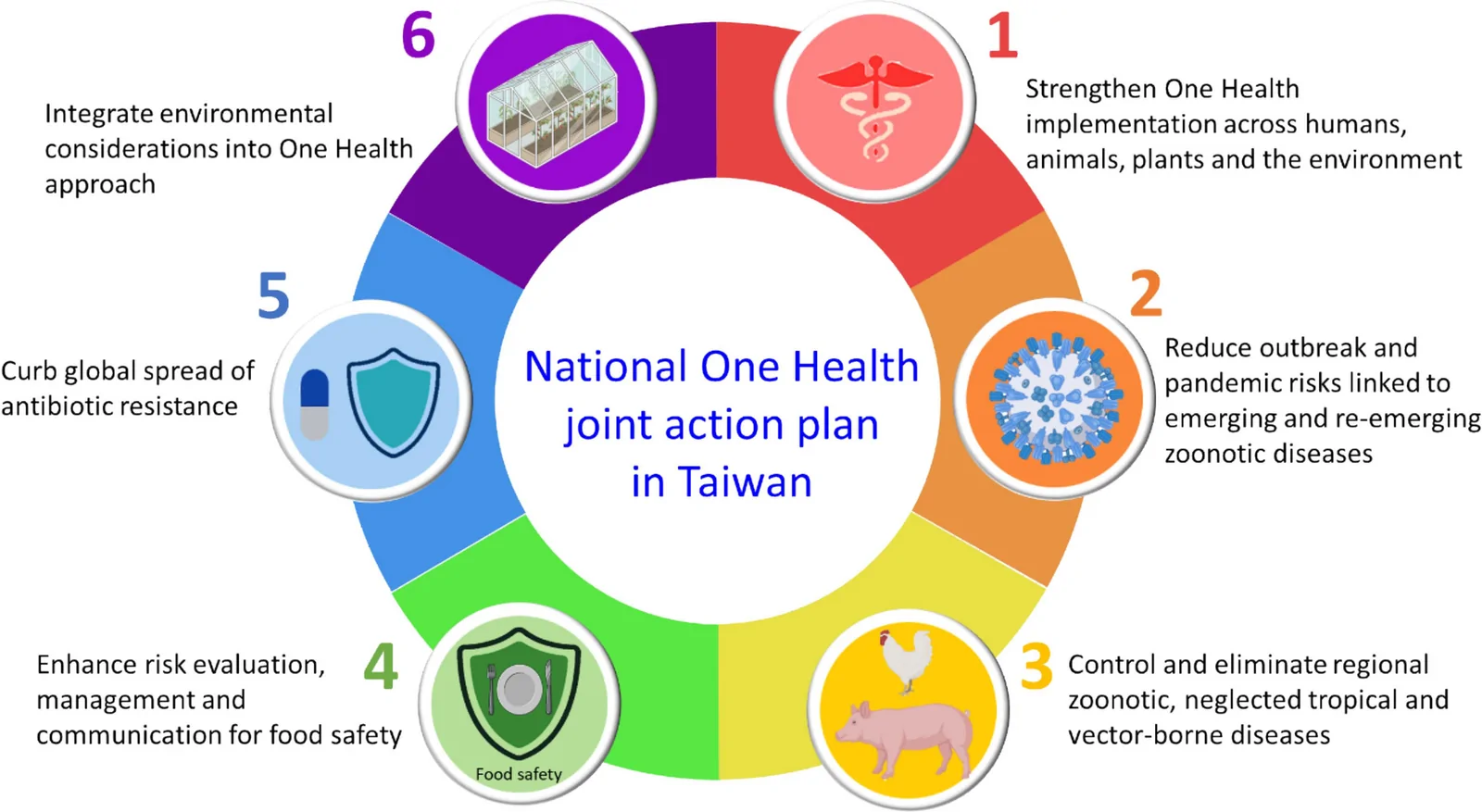
National One Health joint action plan strategic objectives in Taiwan. A schematic summary of the National One Health joint action plan showing six prioritized strategic objectives that guide cross-sectoral coordination across human, animal, plant, and environmental health to prevent and manage zoonotic threats and food safety risks. This framework reflects the collaborative strategy among governmental, medical, agricultural, and environmental sectors to enhance national preparedness, early warning, and coordinated response to zoonotic and environmental health threats. The schematic diagram was created by the authors using BioRender.com (BioRender, Toronto, ON, Canada). Created in BioRender by Lin (2026). https://BioRender.com/n8c6tsl

The National One Health Joint Action Plan (2026–2030) is organized around 6 strategic tracks: governance, emerging zoonoses, vector-borne diseases, food-safety risk management, AMR; and environmental integration (Fig. 3). For influenza and other zoonoses, operational priorities emphasize integration of public-health, veterinary, and environmental data streams for early warning, with particular focus on highly pathogenic avian influenza and swine influenza A viruses. Community risk management is strengthened for vector-borne threats in Taiwan’s subtropical context and high-mobility settings. The plan further prioritizes deployment of rapid diagnostics, ecological risk assessment for food safety and AMR control, and development of an interdisciplinary workforce spanning biomedical, veterinary, and ecological sciences.

- **Strengthen One Health implementation across humans, animals, plants, and the environment:** The principle focuses on integrating institutional frameworks and establishing cross-sectoral governance at the national level. It aims to enhance public-health security by applying internationally recognized assessment tools and harmonized operating procedures.
- **Reduce outbreak and pandemic risks linked to emerging and re-emerging zoonotic diseases:** This track seeks to integrate surveillance and response for priority zoonoses, expand rapid-response capacity, cultivate interdisciplinary expertise, and promote targeted R&D to upgrade epidemic prevention across sectors.
- **Control and eliminate regional zoonotic, neglected tropical, and vector-borne diseases:** Key actions include developing and maintaining a surveillance platform for blood-feeding vectors and conducting seroepidemiological studies to refine burden estimates. The track further strengthens risk assessment and monitoring of vector- and environment-related diseases.
- **Enhance risk evaluation, management, and communication for food safety:** This track promotes continuous monitoring of food-production processes and microbial risks. Artificial intelligence is used for border inspection to reinforce import safety protocols and align domestic regulations with international standards.
- **Curb the global spread of antimicrobial resistance:** Core elements include strengthening interdisciplinary AMR surveillance networks, adopting innovative AI-enabled analytics, and integrate surveillance of antibiotic usage and supply chains to inform stewardship policies.
- **Integrate environmental considerations into the One Health approach:** This track aims to mainstream pollution control and ecological risk management into health planning. The plan seeks to safeguard biodiversity and food security as integral components of public-health resilience.

Collectively, these measures aim to translate One Health principles into operational capacity. By aligning governance, surveillance, workforce development, and technological innovation, the plan seeks to reduce zoonotic risk and enhance national and regional pandemic preparedness.

## Emerging technologies for influenza surveillance in Taiwan

Over the past two decades Taiwan’s influenza surveillance has evolved from manual, event-driven reporting to a multilayered digital system. This system integrates animal, human, and environmental components under a One Health governance model. BAPHIQ and AHRI coordinate farm- and slaughterhouse-based animal surveillance. TCDC oversees sentinel and laboratory networks for humans, and population-scale metadata from the NHI database supports epidemiological inference. Taiwan contributes human and animal sequences to GISAID and shares animal-health intelligence through OFFLU. These works align national practice with global influenza-response systems (Yang et al. 2025).

### Veterinary, clinical, and environmental surveillance

A robust network requires standardized, representative sampling across complementary streams. Taiwan’s influenza surveillance operates through 3 coordinated streams:

- **Veterinary field sampling:** Sentinel swine farms and slaughterhouses provide continuous specimens for virus isolation and molecular testing. Routine specimens include nasal and tracheal swabs, bronchoalveolar lavage fluid, and lung tissue from symptomatic animals. Quarterly active sampling by BAPHIQ targets at least 30 animals per site across northern, central, and southern regions. This approach helps detect geographically restricted reassortants and emerging lineages (MOA 2022b). Animal surveillance is linked to productivity metrics and movement histories to support epidemiologic investigation and traceability. Although this review focuses primarily on swine influenza surveillance, the same One Health architecture could be extended to other zoonotic influenza threats, including highly pathogenic H5 viruses and G1-lineage H9N2 viruses. Such expansion would require host-specific surveillance streams, including poultry and wild-bird sampling, environmental monitoring at poultry farms, live-bird markets, wetlands, and migratory-bird interfaces, and targeted surveillance of occupationally exposed human populations (Yang et al. 2017). These avian influenza streams should remain operationally distinct from swine surveillance but could be integrated through shared genomic sequencing, interagency data exchange, and joint risk assessment. This approach would allow Taiwan’s One Health framework to support broader zoonotic influenza preparedness while preserving clear distinctions among swine, avian, and human virus–host systems.
- **Human clinical sampling:** TCDC’s sentinel hospitals and outpatient clinics collect nasopharyngeal swabs from patients with ILI within 72 h of symptom onset. Enhanced sampling among occupationally exposed groups (farmers, veterinarians, and slaughterhouse workers) helps the detection of potential cross-species infections (TCDC 2014, 2025b). Vaccination-uptake, lot-specific adverse-event, and visit-level metadata are integrated through the NHI smart-card system and NIIS. These data support vaccine-effectiveness and safety assessments.
- **Environmental sampling:** Environmental surveillance, a recent addition, includes wastewater and abattoir-effluent testing for influenza RNA. Pilot data indicate environmental viral loads often rise about one week before clinical ILI spikes. This finding supports wastewater surveillance as a cost-effective early-warning layer (Chou et al. 2025). Environmental monitoring is increasingly incorporated into situational awareness dashboards to complement clinical and veterinary streams.

### Genomic monitoring and animal disease prevention

Next-generation sequencing (NGS) has transformed Taiwan’s molecular surveillance capacity. NHRI and partner laboratories deploy Illumina and Oxford Nanopore platforms for whole-genome sequencing of field isolates. Automated bioinformatic pipelines assemble the 8 viral segments and annotating coding regions (NHRI 2024). Sequence data are uploaded to GISAID and visualized via Microreact and Nextstrain. These tools contextualize phylogenetic relationships and gene-flow between humans and swine, e.g., A/Taiwan/1/2021 (H1N2v).

To accelerate decision making, smart sequencing workflows flag unusual segment constellations and antigenic-site substitutions in near real time, enabling rapid risk triage. BAPHIQ’s Animal Disease Prevention Information Network, specimen barcoding, and linked farm-slaughter inspection systems streamline specimen tracking and shorten laboratory turnaround times, while serum testing at designated livestock research institutes supports sero-epidemiologic inference and farm-level disease prevention (BAPHIQ 2018, 2025).

### Data interoperability and visualization

Interoperability among clinical, veterinary, and genomic datasets is central to timely risk assessment. Taiwan is implementing FHIR-based standards to harmonize electronic records across human, animal, and environmental systems (NHIA 2025a). Interactive dashboards visualize viral activity by subtype, species, and region to support joint situational awareness. Computable clinical rules (e.g., Clinical Quality Language) are being developed as a national rule library. They encode escalation triggers, automate notifications across TCDC–BAPHIQ–AHRI workflows, and standardize evidence-based responses while preserving auditability and version control (NHIA 2025b). Field-deployable molecular assays (LAMP) and CRISPR-Cas diagnostics enable on-farm subtype identification. Smartphone-linked readers transmit encrypted results to central databases. This creates a real-time surveillance tier valuable for rural and resource-limited settings (Yu et al. 2020). Together, these interoperable platforms support a shift from reactive detection to predictive, data-driven surveillance.

### AI-driven analytics and digital health infrastructure

Taiwan combines AI, big-data analytics, and near-real-time reporting to strengthen early warning and short-term forecasting (Lin et al. 2018). The national information stack (centered on NHI claims and the RODS syndromic platform) supports AI-enhanced nowcasting and short-range forecasts. These systems maintain privacy and audit trails (Esan et al. 2012). Deep-learning models, including LSTM architectures trained on global sequence archives, are being used experimentally (Sim and Mackie 2009). They forecast amino-acid substitutions associated with altered receptor binding or immune escape. Climate-informed models that incorporate temperature and humidity have produced forecasts predicting epidemic onset two to four weeks ahead. These models are most effective when coupled with surveillance data (TCDC 2018b). Automated pipelines that ingest NGS output allow AI algorithms to screen for unusual gene-segment constellations and antigenic-site mutations. These processes trigger early alerts to TCDC and BAPHIQ (TCDC 2025c). The Next-generation digital healthcare platform and SmartEHR Coder project aim to accelerate standardized data extraction and FHIR resource generation. This enables seamless cross-institutional exchange and downstream surveillance queries (TCDC 2024, 2025d). Academic–industry collaborations have translated research prototypes (AI imaging, automated coding, and forecasting stations) into operational tools. These tools are deployed across hospitals and public-health networks. They reinforce Taiwan’s Precision One Health ecosystem and improve diagnostic accuracy, timeliness, and workforce efficiency. These emerging technologies, sequencing, environmental surveillance, interoperable data standards and AI, collectively advance Taiwan’s capacity to detect, characterize, and respond to influenza threats. Continued investment in governance, data quality, workforce training, and ethical safeguards will be essential. These measurements maximize public-health benefit while maintaining privacy and system resilience.

### Implementation challenges and governance constraints

Despite the substantial progress of Taiwan’s influenza surveillance framework, several operational and structural challenges continue to limit the full realization of an integrated One Health system. First, effective surveillance depends on a specialized workforce that spans veterinary diagnostics, field epidemiology, genomic analysis, bioinformatics, environmental monitoring, and public-health coordination. However, these capacities are not always evenly distributed across institutions, and sustained workforce development remains necessary to support routine surveillance, data interpretation, and rapid cross-sector response. Second, although Taiwan has strengthened interagency coordination, interoperability among veterinary, clinical, environmental, and genomic data systems remains a practical challenge. Differences in reporting formats, data architecture, administrative authority, and access procedures may still impede seamless information exchange and slow coordinated risk assessment.

In addition, the long-term sustainability of advanced surveillance components depends on continued financial and political commitment. Routine whole-genome sequencing, environmental monitoring, digital infrastructure maintenance, and AI-assisted analytics all require sustained investment in equipment, technical personnel, data governance, and institutional support. The implementation of One Health surveillance may also be slowed by sector-specific priorities, uneven local capacity, and resistance to workflow redesign across established administrative systems. These barriers highlight that surveillance integration is not solely a technical issue, but also an organizational and governance challenge. Furthermore, the growing use of big-data platforms and AI-enabled analytics introduces important ethical considerations, including privacy protection, data security, algorithmic transparency, and equity in access to advanced surveillance tools. Accordingly, the continued development of Taiwan’s surveillance framework will require not only technological innovation, but also sustained policy refinement, governance adaptation, and investment in institutional resilience.

### Expanding the environmental dimension of One Health surveillance

A further area requiring greater emphasis is the environmental dimension of influenza surveillance (Bruno et al. 2026). Although wastewater surveillance, abattoir effluent testing, and micro-environmental sensing represent important advances, the environmental contribution to influenza risk extends beyond these technical tools alone. Ecological context, land-use structure, farm density, animal movement patterns, waste management practices, and local climatic conditions may all shape viral persistence, transmission opportunities, and the amplification of outbreaks at the animal–environment–human interface. In intensive livestock systems, environmental conditions such as temperature, humidity, ventilation quality, and seasonal fluctuations may influence both viral stability and host exposure dynamics, thereby affecting the timing and scale of transmission (Nazia et al. 2026).

From a One Health perspective, environmental surveillance should therefore not be regarded merely as a supplementary monitoring layer, but as an integral component of risk interpretation and early warning. More systematic incorporation of ecological metadata, environmental exposure indicators, and climate-sensitive analytical models may improve the interpretive value of surveillance data and strengthen zoonotic risk assessment. In Taiwan, where livestock production, human population density, and environmental pressures intersect within a relatively compact geographic setting, further integration of environmental intelligence could enhance the sensitivity and predictive capacity of the national surveillance framework. Future development should therefore move toward a more comprehensive model in which veterinary, clinical, genomic, and environmental signals are jointly analyzed to support earlier detection and more context-aware preparedness.

## Conclusions

Swine influenza A viruses present a dual threat: zoonotic risk to humans and economic disruption to swine production. Taiwan, characterized by dense human–swine interfaces, illustrates how systemic vulnerability can be converted into resilience through integrated surveillance. Over the past two decades the country has shifted from fragmented, reactive reporting to a multilayered One Health ecosystem. This ecosystem unifies hospitalization, virological, outpatient, mortality, and animal surveillance. The 2009 H1N1 pandemic was a catalytic event, revealing structural weaknesses. It also accelerated reforms in notification frameworks, genomic monitoring, and interagency coordination. Taiwan’s current architecture emphasizes precision monitoring. Active farm-based sampling, routine whole-genome sequencing, and prompt WHO/IHR notification enable high-resolution detection of reassortants and zoonotic spillovers. The documented human H1N2v cases (2021–2023) confirm ongoing bidirectional virus exchange. They validate integrated human–animal–environment surveillance. Adoption of AI-enabled animal monitoring, AI-driven forecasting, and wastewater-based epidemiology has improved situational awareness. These tools have reduced the detection delays and optimized resource allocation during epidemic peaks. In comparative perspective, while North America and Europe sustain large-scale, genomics-enabled infrastructures, Taiwan demonstrates that smaller systems can achieve high transparency and rapid reporting. Taiwan also makes meaningful contributions to international networks such as OFFLU and GISAID. Strategic integration, targeted technological deployment, and policy coherence can offset scale limitations. Together they produce outsized public-health returns. The National One Health Joint Plan of Action (2026–2030) institutionalizes cross-sectoral collaboration across health, agriculture, and environment. It aligns national priorities with WHO, FAO, and WOAH frameworks. Embedding One Health principles into governance strengthens Taiwan’s ability to mitigate zoonotic risk, manage food-safety threats, and confront AMR. Taiwan’s trajectory shows that durable pandemic preparedness rests on scientific and technological innovation combined with sustained political commitment and societal engagement. It offers a practical, replicable model for other countries facing the intertwined challenges of zoonoses, agricultural stability, and global health security.

## Data Availability

All data in this study were obtained from published material in scientific journals, referenced in the paper, and can be obtained by any individual with access.

## Abbreviations

AHRI: Animal Health Research Institute of Taiwan
AI: Artificial intelligence
BAPHIQ: Bureau of Animal and Plant Health Inspection and Quarantine of Taiwan
CDC: Centers for Disease Control and Prevention of USA
CECC: Central Epidemic Command Center of Taiwan
ECDC: European Centre for Disease Prevention and Control
EFSA: European Food Safety Authority
EU: European Union
FAO: Food and Agriculture Organization of the United Nations
ICU: Intensive Care Unit
IHR: International Health Regulations
ILI: Influenza-like illness
LAMP: Loop-mediated isothermal amplification
MOA: Ministry of Agriculture of Taiwan
NAHLN: National Animal Health Laboratory Network
NHIA: National Health Insurance Administration of Taiwan
NIIS: National Immunization Information System of Taiwan
OFFLU: OIE/FAO Network of expertise on animal influenza
OHHLEP: One Health High-Level Expert Panel
RFID: Radio frequency identification
RODS: Real-time outbreak and disease surveillance
TCDC: Taiwan Centers for Disease Control
USDA: United States Department of Agriculture
WHO: World Health Organization
WHO/IHR: World Health Organization / International Health Regulations

## References

[CR1] Amato Gauci AJ, Zucs P, Snacken R (2010) The 2009 A(H1N1) pandemic in Europe: a review of the experience. European Centre for Disease Prevention and Control, Stockholm. http://ecdc.europa.eu/en/publications/Publications/101108_SPR_pandemic_experience.pdf. Accessed 31 Oct 2025

[CR2] Animal Health Research Institute of Taiwan (AHRI) (2018) Epidemic prevention and quarantine guidelines. https://www.aphia.gov.tw/ws.php?id=17908. Accessed 31 Oct 2025

[CR3] Animal Health Research Institute of Taiwan (ARHI) (2021) Quarantine Cloud single window service platform. https://cloudapq.aphia.gov.tw/login. Accessed 31 Oct 2025

[CR4] Assavacheep P, Thanawongnuwech R (2022) Porcine respiratory disease complex: dynamics of polymicrobial infections and management strategies after the introduction of the African swine fever. Front Vet Sci 9:1048861. 10.3389/fvets.2022.1048861 36504860 PMC9732666

[CR5] Bronzwaer S, Catchpole M, de Coen W, Dingwall Z, Fabbri K, Foltz C, Ganzleben C, van Gorcom R, Humphreys A, Jokelainen P, Liebana E, Rizzi V, Url B (2022) One health collaboration with and among EU agencies—bridging research and policy. One Health 15:100464. 10.1016/j.onehlt.2022.100464 36561708 PMC9767809

[CR6] Bruno L, Nappo MA, Frontoso R, Montinaro S, Di Lecce R, Guarnieri C, Ferrari L, Corradi A (2026) Avian influenza viruses: global panzootic, host range expansion and emerging one-health threats. Vet Sci 13(1):67. 10.3390/vetsci13010067 41600723 PMC12846701

[CR7] Bureau of Animal and Plant Health Inspection and Quarantine of Taiwan (BAPHIQ) (2018) Annual report. https://www.aphia.gov.tw/Publish/20190815/files/assets/common/downloads/publication.pdf. Accessed 31 Oct 2025

[CR8] Bureau of Animal and Plant Health Inspection and Quarantine of Taiwan (BAPHIQ) (2025) Animal and plant health inspection and quarantine information. https://www.aphia.gov.tw/ws.php?id=12498. Accessed 31 Oct 2025

[CR9] Centers for Disease Control of USA (CDC) (2010) 2009 H1N1 pandemic (H1N1pdm09 virus). https://archive.cdc.gov/www_cdc_gov/flu/pandemic-resources/2009-h1n1-pandemic.html. Accessed 31 Oct 2025

[CR10] Centers for Disease Control of USA (CDC) (2025) About One Health. https://www.cdc.gov/one-health/about/index.html. Accessed 31 Oct 2025

[CR11] Chepkwony S, Parys A, Vandoorn E, Stadejek W, Xie J, King J, Graaf A, Pohlmann A, Beer M, Harder T, Van Reeth K (2021) Genetic and antigenic evolution of H1 swine influenza A viruses isolated in Belgium and the Netherlands from 2014 through 2019. Sci Rep 11:11276. 10.1038/s41598-021-90512-z 34050216 PMC8163766

[CR12] Chou HW, Wang CH, Lin LY, Chi NH, Chou NK, Yu HY, Chen YS (2020) Prognostic factors for heart recovery in adult patients with acute fulminant myocarditis and cardiogenic shock supported with extracorporeal membrane oxygenation. J Crit Care 57:214–219. 10.1016/j.jcrc.2020.03.007 32220770

[CR13] Chou YC, Lin FH, Hsieh CJ, Yu CP (2025) Increased risk of influenza-like illness clusters in schools, Taiwan from 2011 to 2020: a retrospective study. J Epidemiol Glob Health 15(1):16. 10.1007/s44197-025-00366-1 39910014 PMC11799474

[CR14] Chuang JH, Huang AS, Huang WT, Liu MT, Chou JH, Chang FY, Chiu WT (2012) Nationwide surveillance of influenza during the pandemic (2009–10) and post-pandemic (2010–11) periods in Taiwan. PLoS ONE 7(4):e36120. 10.1371/journal.pone.0036120 22545158 PMC3335813

[CR15] de Courville C, Cadarette SM, Wissinger E, Alvarez FP (2022) The economic burden of influenza among adults aged 18 to 64: a systematic literature review. Influenza Other Respir Viruses 16(3):376–385. 10.1111/irv.12963 35122389 PMC8983919

[CR16] Er JC (2025) Improving influenza nomenclature based on transmission dynamics. Viruses 17(5):633. 10.3390/v17050633 40431645 PMC12115919

[CR17] Esan O, Cowin A, Olowokure B (2012) Influenza A(H1N1)pdm09 and flu response centres: characteristics of flu response centre staff in the West Midlands. Public Health 126(9):804–809. 10.1016/j.puhe.2012.06.008 22929234 PMC7118750

[CR18] Food and Agriculture Organization of the United Nations (FAO) (2024a) Meat market review. https://openknowledge.fao.org/server/api/core/bitstreams/0d3971d1-2fba-4381-8bac-b3a251ee716a/content. Accessed 31 Oct 2025

[CR19] Food and Agriculture Organization of the United Nations (FAO) (2024b) One Health definition and principles. https://www.fao.org/one-health/highlights/highlights-detail/one-health-definition-and-principles/en. Accessed 31 Oct 2025

[CR20] Gong L, Xu R, Wang Z, Deng Q, Wang H, Zhang G (2018a) African swine fever recovery in China. Vet Med Sci 6:890–893. 10.1002/vms3.299 PMC7738741 32602251

[CR21] Gong YN, Kuo RL, Chen GW, Shih SR (2018b) Centennial review of influenza in Taiwan. Biomed J 41(4):234–241. 10.1016/j.bj.2018.08.002 30348266 PMC6197989

[CR22] Hsueh PR, Lee PI, Chiu AWH, Yen MY (2010) Pandemic (H1N1) 2009 vaccination and class suspensions after outbreaks, Taipei City, Taiwan. Emerg Infect Dis 16(8):1309–1311. 10.3201/eid1608.100310 20678333 PMC3298312

[CR23] Hui X, Tian X, Ding S, Gao G, Cui J, Zhang C, Zhao T, Duan L, Wang H (2025) A review of cross-species transmission mechanisms of influenza viruses. Vet Sci 12(5):447. 10.3390/vetsci12050447 40431540 PMC12115712

[CR24] Kao TM, Wang CH, Chen YC, Ko KW, Chang SC (2009) The first case of severe novel H1N1 influenza successfully rescued by extracorporeal membrane oxygenation in Taiwan. J Formos Med Assoc 108(11):894–898. 10.1016/S0929-6646(09)60422-8 19933034

[CR25] Korsun N, Trifonova I, Pavlova D, Uzunova Y, Ivanov I, Ivanov D, Velikov P, Voleva S, Tcherveniakova T, Christova I (2025) Etiological spectrum of acute respiratory infections in Bulgaria during the 2023–2024 season and genetic diversity of circulating influenza viruses. Viruses 17(2):270. 10.3390/v17020270 40007025 PMC11860199

[CR26] Kristensen C, Larsen LE, Trebbien R, Jensen HE (2024) The avian influenza A virus receptor SA-α2,3-Gal is expressed in the porcine nasal mucosa sustaining the pig as a mixing vessel for new influenza viruses. Virus Res 340:199304. 10.1016/j.virusres.2023.199304 38142890 PMC10793167

[CR27] Lai SK, Chang HL, Wu HS, Chuang JH (2010) Multiple disease surveillance systems against pandemic (H1N1) influenza in Taiwan. Taiwan Epidemiology Bulletin. https://www.cdc.gov.tw/En/EpidemicTheme/Detail/hQ4XhaZAzUmNe2ksY4tjMA?archiveId=PM23Fx6v5jODQ0JR0j4BWg. Accessed 31 Oct 2025

[CR28] Li WC, Chang CY, Liu YP, Chen LH, Gong YN, Lin YJ, Chen GW, Shih SR (2025) Intensive reassortment and frequent intercontinental transmission revealed by long-term genetic analysis of H10 avian influenza viruses in Taiwan. Emerg Microbes Infect 14(1):2556794. 10.1080/22221751.2025.2556794 40966411 PMC12447466

[CR29] Liang W, Feng L, Xu C, Xiang N, Zhang Y, Shu Y, Wang H, Luo H, Yu H, Liang X, Li D, Lee CK, Feng Z, Hou Y, Wang Y, Chen Z, Yang W (2012) Response to the first wave of pandemic (H1N1) 2009: experiences and lessons learnt from China. Public Health 126(5):427–436. 10.1016/j.puhe.2012.02.008 22516790 PMC7111655

[CR30] Lin CH (2026) Swine Influenza Surveillance and One Health Governance in Taiwan Created in BioRender. https://BioRender.com/n8c6tsl. Accessed 30 June 2026

[CR31] Lin LY, Warren-Gash C, Smeeth L, Chen PC (2018) Data resource profile: the National Health Insurance Research Database (NHIRD). Epidemiol Health 40:e2018062. 10.4178/epih.e2018062 30727703 PMC6367203

[CR32] Mancera Gracia JC, Pearce DS, Masic A, Balasch M (2020) Influenza A virus in swine: epidemiology, challenges and vaccination strategies. Front Vet Sci 7:647. 10.3389/fvets.2020.00647 33195504 PMC7536279

[CR33] Ministry of Agriculture of Taiwan (MOA) (2002) Current status of implementation of foot-and-mouth disease eradication measures. https://www.moa.gov.tw/ws.php?id=4276. Accessed 31 Oct 2025

[CR34] Ministry of Agriculture of Taiwan (MOA) (2022a) History and business introduction. https://www.nvri.gov.tw/Module/DisplayPageContent.aspx?pid=ZHoOioo/2FrMU/3. Accessed 31 Oct 2025

[CR35] Ministry of Agriculture of Taiwan (MOA) (2022b) 5G technology adds value to new agriculture—A new type of IoT for livestock and poultry health and disease prevention. https://www.moa.gov.tw/theme_data.php?theme=news&sub_theme=agri&id=8648. Accessed 31 Oct 2025

[CR36] Ministry of Agriculture of Taiwan (MOA) (2025) Agricultural internet of things development plan. https://join.gov.tw/acts/detail/e9c4b081-d459-4b57-8110-af2b7c013713. Accessed 31 Oct 2025

[CR37] Ministry of Health and Welfare (2025) National One Health Joint Action Plan. Executive Yuan of Taiwan. https://english.ey.gov.tw/News3/9E5540D592A5FECD/d8de66c5-4370-4083-a455-f1d7cf1a25fa. Accessed 31 Oct 2025

[CR38] Moraes D, Cezar G, Magalhaes E, Trevisan G, Linhares D (2022) Information for swine influenza A virus (IAV) RT-PCR detection from porcine cases is now available on the monthly PDF reports and online SDRS dashboards. Swine Disease Report System, Report No. 50:7–8. https://www.fieldepi.org/SDRS. Accessed 31 Oct 2025

[CR39] Moraes DCA, L Vincent Baker A, Wang X, Zhu Z, Berg E, Trevisan G, Zhang J, Jayaraman S, Linhares DCL, Gauger PC, S Silva G (2023) Veterinarian perceptions and practices in prevention and control of influenza virus in the Midwest United States swine farms. Front Vet Sci 10:1089132. 10.3389/fvets.2023.1089132 PMC9936088 36816189

[CR40] National Health Insurance Administration of Taiwan (NHIA) (2025a) Taiwan NHI clinical quality language implementation guide. https://build.fhir.org/ig/TWNHIFHIR/cql/index.html. Accessed 31 Oct 2025

[CR41] National Health Insurance Administration of Taiwan (NHIA) (2025b) Launch meeting of the Taiwan medical information standards platform of the Ministry of Health and Welfare. https://www.mohw.gov.tw/cp-16-81833-1.html. Accessed 31 Oct 2025

[CR42] National Health Research Institutes of Taiwan (NHRI) (2024) Record of the inauguration ceremony and academic activities of the “Health Infectious Diseases Bank Nanopore Centre of Excellence in National Health Research Institutes”. https://nidb.nhri.edu.tw/p-20240410.html. Accessed 31 Oct 2025

[CR43] Nazia N, Pullenayegum E, Loeb M (2026) Individual and environmental factors influencing influenza transmission: a multilevel analysis. Influenza Other Respir Viruses 20(2):e70232. 10.1111/irv.70232 41636348 PMC12869838

[CR44] One Health High-Level Expert Panel (OHHLEP), Adisasmito WB, Almuhairi S, Behravesh CB, Bilivogui P, Bukachi SA, Casas N, Cediel Becerra N, Charron DF, Chaudhary A, CiacciZanella JR, Cunningham AA, Dar O, Debnath N, Dungu B, Farag E, Gao GF, Hayman DTS, Khaitsa M, Koopmans MPG, Machalaba C, Mackenzie JS, Markotter W, Mettenleiter TC, Morand S, Smolenskiy V, Zhou L (2022) One Health: a new definition for a sustainable and healthy future. PLoS Pathog 18(6):e1010537. 10.1371/journal.ppat.1010537 35737670 PMC9223325

[CR45] Republic of China Swine Association (2025) Current status of the domestic swine industry. https://www.tafah.org.tw/download_file_1_73.htm. Accessed 31 Oct 2025

[CR46] Roy S, Hassan MM, Mohd G, Rizwan AS, Sendhilkumar M, Lydia JB, Priya J, Ponnaiah M, Wijesinghe PR, Salvador EC, Buddha N, Magalhaes RS, Kakkar M, Murhekar M (2025) Implications for influenza A virus surveillance in Southeast Asian region countries: a scoping review of approaches for the surveillance of swine influenza viruses at human–swine interfaces. BMJ Public Health 3(1):e002330. 10.1136/bmjph-2024-002330 40548069 PMC12182126

[CR47] Sim F, Mackie P (2009) Pandemic or no pandemic: emergence of swine influenza A (H1N1) in 2009. Public Health (Lond) 123(6):405–406. 10.1016/j.puhe.2009.05.006 19539828

[CR48] Stern AM, Cetron MS, Markel H (2010) The 1918–1919 influenza pandemic in the United States: lessons learned and challenges exposed. Public Health Rep 125(Suppl 3):6–8. 10.1177/00333549101250S303 20568564 PMC2862329

[CR49] Taipei Times (2022) Girl confirmed as second case of swine flu variant. https://www.taipeitimes.com/News/taiwan/archives/2022/12/07/2003790268. Accessed 31 Oct 2025

[CR50] Taiwan Centers for Disease Control (TCDC) (2009a) First severe case of pandemic influenza A (H1N1) confirmed. https://www.cdc.gov.tw/En/Bulletin/Detail/EEvtRGFr40OmbjMFmX67LQ?typeid=158. Accessed 31 Oct 2025

[CR51] Taiwan Centers for Disease Control (TCDC) (2009b) Central Epidemic Command Center notified death in six-year-old girl with pandemic influenza A (H1N1) infection by hospital. https://www.cdc.gov.tw/En/Bulletin/Detail/R_mRdu1HsPZHQqPeG6p_WA?typeid=158. Accessed 31 Oct 2025

[CR52] Taiwan Centers for Disease Control (TCDC) (2009c) Taiwan’s response to the H1N1 influenza. https://www.cdc.gov.tw/uploads/files/4c6e5d73-12a1-4b7a-b9a1-486c0c118629.pdf. Accessed 31 Oct 2025

[CR53] Taiwan Centers for Disease Control (TCDC) (2009d) Real-time surveillance of pneumonia and influenza mortalities via the national death certificate system. https://www.cdc.gov.tw/En/EpidemicTheme/Detail/hQ4XhaZAzUmNe2ksY4tjMA?archiveId=OTg3aKgFKvIU2i_O0j65dQ. Accessed 31 Oct 2025

[CR54] Taiwan Centers for Disease Control (TCDC) (2012) Brief introduction to my country’s four major surveillance systems for H1N1 novel influenza. https://www.cdc.gov.tw/Category/ListContent/81xBc8ZjnReHKsB55Jhezg?uaid=YN3QqJSwNrf0oFHYW3cbSw. Accessed 31 Oct 2025

[CR55] Taiwan Centers for Disease Control (TCDC) (2014) National immunization information system (NIIS). https://www.cdc.gov.tw/Category/Page/z_vOjN1F__1odOkk2vI5cA. Accessed 31 Oct 2025

[CR56] Taiwan Centers for Disease Control (TCDC) (2016) Taiwan influenza express (2016/03/06–2016/03/12). https://www.cdc.gov.tw/info.aspx?treeid=1F07E8862BA550CF&nowtreeid=204B424A955D572F&tid=C363651DDC5981F3. Accessed 31 Oct 2025

[CR57] Taiwan Centers for Disease Control (TCDC) (2018a) Taiwan influenza express (2018/03/04–2018/03/10). https://www.cdc.gov.tw/info.aspx?treeid=1F07E8862BA550CF&nowtreeid=CB122F160E7AC2B9&tid=3D8AA6E7A9B58EDA. Accessed 31 Oct 2025

[CR58] Taiwan Centers for Disease Control (TCDC) (2018b) The Taiwan Centers for Disease Control has partnered with Acer to launch an AI-powered flu forecasting station, enabling real-time monitoring of future epidemic developments. https://www.cdc.gov.tw/Category/ListContent/AHwuigegBBBmuDcbWkzoGQ?uaid=f9nXXPLjkHuC-lE0V4s8Wg. Accessed 31 Oct 2025

[CR59] Taiwan Centers for Disease Control (TCDC) (2022) The second case of H1N2v influenza virus isolated from a patient with respiratory disease. https://www.cdc.gov.tw/Bulletin/Detail/5hCiiinlbOGZjvdYMKXdYA?typeid=9. Accessed 31 Oct 2025

[CR60] Taiwan Centers for Disease Control (TCDC) (2023) The third case of H1N2v influenza virus isolated from a patient with respiratory disease. https://www.cdc.gov.tw/Bulletin/Detail/0_iopaGC4g9Grsmb602QCg?typeid=9. Accessed 31 Oct 2025

[CR61] Taiwan Centers for Disease Control (TCDC) (2024) Next-generation digital healthcare platform plan. https://blog.mohw.gov.tw/459/. Accessed 31 Oct 2025

[CR62] Taiwan Centers for Disease Control (TCDC) (2025a) Annual influenza vaccination program. https://www.cdc.gov.tw/Category/MPage/JNTC9qza3F_rgt9sRHqV2Q. Accessed 31 Oct 2025

[CR63] Taiwan Centers for Disease Control (TCDC) (2025b) Application of next-generation sequencing in public health—detection of Naegleria fowleri by using metagenomic sequencing. https://www.cdc.gov.tw/File/Get/7KjTc2-T_B5PYNt2g5Z8jw. Accessed 31 Oct 2025

[CR64] Taiwan Centers for Disease Control (TCDC) (2025c) Taiwan CDC organizes One Health workshop to formulate national One Health joint plan of action. https://www.cdc.gov.tw/En/Bulletin/Detail/eXozIOKkwu0AJvYmjQZCjw?typeid=158. Accessed 31 Oct 2025

[CR65] Taiwan Centers for Disease Control (TCDC) (2025d) The Ministry of Health and Welfare’s design of an automatic coding assistance system, which contributes to the standardization of electronic medical records in Taiwan, won the Future Technology Award. https://www.mohw.gov.tw/cp-16-84374-1.html. Accessed 31 Oct 2025

[CR66] Thomas MN, Janzen GM, Markin A, Sharma A, Hewitt K, Li G, Baker AL, Gauger PC, Anderson TK (2025) Active surveillance for influenza A virus in swine reveals within-farm reassortment and cocirculation of distinct subtypes and genetic clades. Vet Microbiol 309:110681. 10.1016/j.vetmic.2025.110681 40818301 PMC12380368

[CR67] Trevisan G, Schwartz KJ, Burrough ER, Arruda B, Derscheid RJ, Rahe MC, Magalhães ES, Almeida MN, Main RG, Linhares DCL (2021) Visualization and application of disease diagnosis codes for population health management using porcine diseases as a model. J Vet Diagn Invest 33(3):428–438. 10.1177/1040638721995782 33719758 PMC8120091

[CR68] Trinh TT, Duong BT, Nguyen ATV, Tuong HT, Hoang VT, Than DD, Nam SJ, Sung HW, Yun KJ, Yeo SJ, Park H (2020) Emergence of novel reassortant H1N1 avian influenza viruses in Korean wild ducks in 2018 and 2019. Viruses 13(1):30. 10.3390/v13010030 33375376 PMC7823676

[CR69] Tsai CP, Pan MJ (2003) New H1N2 and H3N1 influenza viruses in Taiwanese pig herds. Vet Rec 153(13):408. 10.1136/vr.153.13.408 14567672

[CR70] Wang TH, Wei KC, Hsiung CA, Maloney SA, Eidex RB, Posey DL, Chou WH, Shih WY, Kuo HS (2007) Optimizing severe acute respiratory syndrome response strategies: lessons learned from quarantine. Am J Public Health 97(Suppl 1):S98–S100. 10.2105/AJPH.2005.082115 17413071 PMC1855001

[CR71] World Health Organization (WHO) (2022) One Health. https://www.who.int/health-topics/one-health#tab=tab_1. Accessed 31 Oct 2025

[CR72] Yang JR, Teng HJ, Liu MT, Li SY (2017) Taiwan’s public health national laboratory system: success in influenza diagnosis and surveillance. Health Secur 15(2):154–164. 10.1089/hs.2016.0104 28418742 PMC5404250

[CR73] Yang JR, Hsu SZ, Kuo CY, Huang HY, Huang TY, Wang HC, Liu MT (2018) An epidemic surge of influenza A(H3N2) virus at the end of the 2016–2017 season in Taiwan with an increased viral genetic heterogeneity. J Clin Virol 99–100:15–21. 10.1016/j.jcv.2017.12.012 29278832

[CR74] Yang H, Gao Q, Zhan B, Cao G, Yu Z (2025) Epidemiological characteristics of influenza after COVID-19 pandemic in Zhejiang Province, China. BMC Infect Dis 25(1):1090. 10.1186/s12879-025-11514-0 40890642 PMC12403933

[CR75] Yen MY, Chiu AW, Schwartz J, King CC, Lin YE, Chang SC, Armstrong D, Hsueh PR (2014) From SARS in 2003 to H1N1 in 2009: lessons learned from Taiwan in preparation for the next pandemic. J Hosp Infect 87(4):185–193. 10.1016/j.jhin.2014.05.005 24996515 PMC7114835

[CR76] Yu LS, Chou SY, Wu HY, Chen YC, Chen YH (2020) Rapid and semi-quantitative colorimetric loop-mediated isothermal amplification detection of ASFV via HSV color model transformation. J Microbiol Immunol Infect 54(5):963–970. 10.1016/j.jmii.2020.08.003 32868194

[CR77] Yu H, Wang Q, Qi B, Qian J, Yang W, Feng L (2025) Changing epidemiological pattern and higher disease burden of influenza in China, 2022 to 2025. Influenza Other Respir Viruses 19(9):e70151. 10.1111/irv.70151 40884275 PMC12397873

